# The metabolic response of the *Bradypus* sloth to temperature

**DOI:** 10.7717/peerj.5600

**Published:** 2018-09-19

**Authors:** Rebecca Naomi Cliffe, David Michael Scantlebury, Sarah Jane Kennedy, Judy Avey-Arroyo, Daniel Mindich, Rory Paul Wilson

**Affiliations:** 1 Swansea Lab for Animal Movement, Biosciences, College of Science, Swansea University, Swansea, Wales, United Kingdom; 2 The Sloth Sanctuary of Costa Rica, Limon, Costa Rica; 3 Research Center, The Sloth Conservation Foundation, Preston, Lancashire, United Kingdom; 4 School of Biological Sciences, Institute for Global Food Security, Queen’s University Belfast, Belfast, Northern Ireland

**Keywords:** Bradypus, Energetics, Arboreal folivore, Metabolic depression, Metabolic rate, Sloth, Temperature response

## Abstract

Poikilotherms and homeotherms have different, well-defined metabolic responses to ambient temperature (*T*_*a*_), but both groups have high power costs at high temperatures. Sloths (*Bradypus*) are critically limited by rates of energy acquisition and it has previously been suggested that their unusual departure from homeothermy mitigates the associated costs. No studies, however, have examined how sloth body temperature and metabolic rate vary with *T*_*a*_. Here we measured the oxygen consumption (VO_2_) of eight brown-throated sloths (*B. variegatus*) at variable *T*_*a*_’s and found that VO_2_ indeed varied in an unusual manner with what appeared to be a reversal of the standard homeotherm pattern. Sloth VO_2_ increased with *T*_*a*_, peaking in a metabolic plateau (nominal ‘thermally-active zone’ (TAZ)) before decreasing again at higher *T*_*a*_ values. We suggest that this pattern enables sloths to minimise energy expenditure over a wide range of conditions, which is likely to be crucial for survival in an animal that operates under severe energetic constraints. To our knowledge, this is the first evidence of a mammal provisionally invoking metabolic depression in response to increasing *T*_*a*_’s, without entering into a state of torpor, aestivation or hibernation.

## Background

In order to survive, animals must remain in positive energy balance over their lifetime, with energy acquisition occurring via food, and energy expenditure occurring via movement (Nathan et al., 2008; Shepard et al., 2013), growth (including tissue regeneration) (Careau et al., 2013; Pontzer et al., 2014), reproduction (Gittleman & Thompson, 1988; Thometz et al., 2016), and physiological homeostasis (Haim & Borut, 1981; Silva, 2005). Temperature regulation has been subject to particular interest within the scientific community because variations in environmental temperature are clear stressors that can be measurable.

Ambient temperature (*T*_*a*_) affects poikilotherms and homeotherms (Schmidt-Nielsen, 1997) in profoundly different ways. Temperature affects the rates of biochemical and enzymatic reactions (Daniel et al., 2010) and it is this thermodynamic effect that ties the performance of poikilotherms, which are unable to internally modulate their core body temperature (*T*_*b*_) independently of their surrounds, to thermal fluctuations in the environment (Schulte, 2015). Instead, they utilise behavioural methods of thermoregulation with the thermal optimum considered to be the ambient (and body) temperature at which metabolic rate is highest and performance optimised (Boyles et al., 2011). Typically poikilotherm metabolic rate rises with *T*_*a*_ to a point where excessive heat causes system breakdown, eventually leading to death (Angilletta, 2009; Huey & Stevenson, 1979). By contrast, homeotherms usually use adaptive thermogenesis to maintain high, stenothermal, *T*_*b*_’s that are largely independent of their surroundings (Lowell & Spiegelman, 2000), maintaining physical performance at a range of *T*_*a*_’s (Pat, Stone & Johnston, 2005). This comes at an energetic cost though (Nagy, 2005), because at low *T*_*a*_’s, where the heat produced by metabolic processes during normal activity does not equal the heat lost (below the thermoneutral zone (TNZ)), animals have to increase their metabolic rate to keep warm (Haim & Borut, 1981). Above the TNZ, homeotherms have to increase metabolic rate to engage in processes that help eliminate excessive heat impinging from the environment (McNab, 2002). This results in the classic ‘U-shaped’ metabolic rate versus temperature curve (Haim & Borut, 1981) with the expectation that homeotherms typically attempt to operate at temperatures within their TNZ in order to minimise energetic costs. When faced with unfavourable conditions or lack of resources(Geiser, 2004; Lovegrove & Génin, 2008; McKechnie & Mzilikazi, 2011) many mammals are capable of invoking a poikilothermic response by entering a state of dormancy such as daily torpor, aestivation or hibernation. During these dormant periods, metabolic rate and body temperature can be depressed for prolonged periods (Wilz & Heldmaier, 2000).

Three-fingered sloths (*Bradypus variegatus*) are not known to enter such states of dormancy, yet are an enigma within the classic poikilotherm-homeotherm groupings. Most particularly, their *T*_*b*_ may fluctuate by up to 10 °C over a 24 h period (Britton & Atkinson, 1938). Anecdotally, they are suggested to behave like reptiles by making behavioural and postural adjustments which are used to control rates of heat gain and loss (Britton & Atkinson, 1938; Huey, 1982; Kearney & Predavec, 2000; Montgomery & Sunquist, 1978). Indeed, there is speculation that this poikilothermic strategy might enable sloths to have the lowest metabolic rates of non-hibernating mammals, some 40–74% of the predicted value relative to body mass (Brody & Lardy, 1946; Geiser, 2004; Irving, Scholander & Grinnell, 1942; McNab, 1978; Nagy & Montgomery, 1980; Pauli et al., 2016). Quite how sloths might respond metabolically to fluctuations in *T*_*a*_ is unknown, in particular given their extraordinary variation in *T*_*b*_.

We hypothesised that, given the strong link between ambient and sloth body temperature (which has led to them being likened to ectotherms), an increase in temperature should, theoretically, result in an increase in metabolic rate. To test this idea we determined the resting metabolic rate (RMR) of eight adult sloths (*B. variegatus*) using indirect calorimetry across a range of *T*_*a*_’s (21–34 °C) while simultaneously recording *T*_*b*_ and documenting postural adjustments. We detail how the sloth metabolic response to variation in *T*_*a*_ appears to be the reverse of that expected for a non-hibernating homeothermic mammal and speculate that this may serve to minimise energy expenditure over a range of environmental conditions.

## Materials and Methods

### Ethics statement

This research was approved by the Swansea University Animal Welfare & Ethical Review Process Group (AWERP), and the Costa Rican government and associated departments (MINAE, SINAC, ACLAC) permit number: R-033-2015. All research was performed in accordance with relevant guidelines and regulations.

### Animals and study site

Eight adult *B. variegatus* sloths (four male, four female) were chosen for metabolic measurements (Table S1). Five of these were captive animals that were wild-born and maintained permanently at the Sloth Sanctuary of Costa Rica (N09°47′56.47″W 082°54′47.20″). The other three sloths were wild-caught and obtained from the protected forested grounds of the Sloth Sanctuary. Wild sloths were caught by hand and were released in the location in which they had been captured following the completion of metabolic rate determination. All experiments were undertaken between 08:00 and 21:00 in the Sloth Sanctuary veterinary clinic between May and September 2015.

### Body temperature (*T*_*b*_) measurements

A miniature temperature logging device (iButton^®^; Thermochron, Dallas Semiconductors, Maxim Integrated Products, Inc., Sunnyvale, CA, USA) (model DS1922L (± 0.0625 °C)) was inserted into the rectum of four sloths using a gloved digit and lubricant. The logger was calibrated prior to use by immersion into a temperature-controlled water bath (Scantlebury et al., 2012) and programmed to record temperature every 30 min. Sloths normally defaecate only once a week, storing faeces in an anal pouch (Gilmore, Da Costa & Duarte, 2001). Rectal insertion of the temperature logger was therefore deemed the least-invasive, non-surgical method of obtaining accurate body temperature values. If faecal pellets were found in the anal pouch of the animal, these were removed prior to logger insertion to ensure the most accurate (and long-term) temperature readings.

### Resting metabolic rate (RMR) measurements

Prior to measurements, all sloths were weighed (E-PRANCE^®^ Portable Hanging Scale (± 0.01 g)). They were then placed in an 87-L Perspex^®^ metabolic chamber (55 cm long × 45 cm high × 35 cm wide). The chamber was placed in a temperature-controlled water bath which was covered with a polystyrene lid. Concrete weights were placed on top of the chamber to prevent it from floating. The water bath (95 cm × 85 cm × 75 cm), also made from Perspex^®^, was lined with black plastic sheeting and supported with an exterior metal frame. Within the metabolic chamber, there was a wooden bar for the animal to hold on to, and from which it could suspend itself upside down. There was a small window in the plastic sheeting (a ‘peep’-hole) through which the sloth could be observed without it being disturbed by the observer.

Oxygen consumption (VO_2_) was measured using an open-flow system with an upstream flow-meter. Fresh air from outside was pumped into the chamber (AIR CADET^®^ Barnant, model 420–1,902 (Barrington Illinois 60010)), via a copper coil submerged in the water bath, at rates of between 6.0 and 12.0 L/min. The flow rate was adjusted to ensure that depressions in oxygen concentration within the chamber remained between 0.2–0.8% (Speakman, 2013). The flow was measured using a flow meter (ICEhte10 platon flow meter 1-12L/min; ICEoxford Limited, Oxford, UK) which was factory calibrated and checked prior to use using a mass-flow generator (Sable Systems Flowkit 100; Las Vegas, NV, USA). The incurrent air flow rate was measured before drying. The system was checked for leaks using a dilute solution of soapy water. The air inlet was located on the opposite side of the chamber to the air outlet to ensure adequate mixing of air within the chamber. Air leaving the chamber was subsampled at 200 ml/min and then dried (using Drierite) before entering an oxygen and carbon dioxide analyser (FoxBox Field Gas Analysis System; Sable Systems International, Las Vegas, NV, USA). The length of tubing leading from the metabolism chamber to the gas analysers was 0.5 m. The lag time for the analyser reading to equilibrate when the tubing was placed into the chamber to subsample the gasses was less than 1 min. The analyser was factory calibrated and set to 20.95% oxygen before each animal was measured. Fresh air readings were recorded at the start and the end of each run to correct for analyser drift. Any drift in the analyser was assumed to be linear for baseline correction. A time of 120 min was allowed at the beginning of each experiment for each sloth to become accustomed to the chamber, for *T*_*b*_ to adjust to the chamber temperature (Figs. S1 and S2) and for the chamber gases to equilibrate (McClune et al., 2015) (Fig. S3). The animals were observed continuously through the peep hole (for welfare reasons) and behaviour and posture noted at four-minute intervals. During measurement periods (i.e., following temperature adjustment periods and when gas concentrations had stabilised), oxygen and carbon dioxide concentrations were recorded manually at two-minute intervals. A total of 15 experimental runs were made: two sloths were tested on three occasions, three sloths were tested on two occasions, and three sloths were tested once (Table S1). An ‘experimental run’ refers to a series of measurements from one animal, taken during the course of a day.

VO_2_ (ml/min) was calculated as:

(1) \begin{eqnarray*}V{O}_{2}= \frac{\mathrm{FR}\cdot ( \left( {\mathrm{F}}_{\mathrm{i}}{\mathrm{O}}_{2}-{\mathrm{F}}_{\mathrm{e}}{\mathrm{O}}_{2} \right) -{\mathrm{F}}_{\mathrm{e}}{\mathrm{O}}_{2}\cdot \left( {\mathrm{F}}_{\mathrm{e}}{\mathrm{CO}}_{2}-{\mathrm{F}}_{\mathrm{i}}{\mathrm{CO}}_{2} \right) )}{(1-{\mathrm{F}}_{\mathrm{e}}{\mathrm{O}}_{2})} \end{eqnarray*}

where FR is the flow rate; F_i_O_2_ is the fractional amount of O_2_in the incurrent air; F_e_O_2_ is the fractional amount of O_2_ in the excurrent air; F_i_CO_2_ is the fractional amount of CO_2_ in the incurrent air; and F_e_CO_2_ is the fractional amount of CO_2_ in the excurrent air (Lighton, 2008). Metabolic rates were calculated using a conversion factor of 20.1 joules per millilitre of oxygen, which is considered correct for an obligate herbivore such as the sloth (Schmidt-Nielsen, 1997). Values for RMR were compared with allometrically predicted values for terrestrial mammals from Kleiber (1961) and White & Seymour (2003).

Thermal conductance, C (ml O_2_/g.h °), was calculated as:

(2) \begin{eqnarray*}C= \frac{{\mathrm{V O}}_{2}}{{T}_{b}-{T}_{a}} \end{eqnarray*}

where VO_2_ is in ml O_2_/g.h, *T*_*b*_ is body temperature (°C), and *T*_*a*_ is ambient temperature (°C) (McNab, 1980).

### Temperature manipulation

The temperature within the chamber was measured at various locations using a copper-constantan thermocouple and monitored on a Tecpel 307P Dual Input Digital Thermometer (± 0.1 °C) (Fig. S4). Chamber temperature was recorded at four-minute intervals throughout the duration of each experimental run.

The first four experimental runs were undertaken with the chamber maintained at constant temperature. The remaining 11 experimental runs had the chamber temperature directly manipulated. Animals were introduced into the metabolic chamber at temperatures marginally lower than the test temperatures (mean 18 °C) for 2 h for complete rectal temperature stabilisation and to allow them some time to habituate to their surroundings before metabolic testing began (see the Supplementary Information for details of preliminary work). Following this, the temperature within the metabolic chamber was increased incrementally in 2-degree steps starting at 21 °C: i.e., 21–23 °C; 23–25 °C; 25–27 °C; 27–29 °C; 29–31 °C; 31-33 °C; 33–34 °C. These temperature brackets were selected as they encompass the extreme range of ambient temperatures to which *Bradypus* sloths are naturally exposed in the wild. In some cases, several RMR readings were made at different temperatures within each temperature bracket (Table S1).

The length of time animals spent at each temperature was sufficient to allow equilibration of gases within the chamber, and for the animal *T*_*b*_ to adjust to the new *T*_*a*_ (Figs. S1–S3). Typically, animals spent 60 min adjusting to each 2-degree temperature increment. Following the c.60-minute adjustment period, when sloths were seen to be at rest and the gas concentrations had stabilised, RMR readings took place and recordings were taken every 2 min for a further 10 min. RMR values were then calculated from the mean of these 5 values. In nearly every case, the sloths were inactive, apart from slow postural adjustments.

Temperatures within the chamber were controlled by varying the temperature of the water bath which contained two electric water heaters (Grant water bath heater circulator) and two water fans which stirred the water in a clockwise direction around the metabolic chamber.

As a control, the empty chamber was taken through 5 different temperature increments on three separate occasions prior to testing with animals. During these control tests, temperatures were recorded from twelve different locations within the chamber (Fig. S4).

### Observations on posture and activity

**Figure 1:**
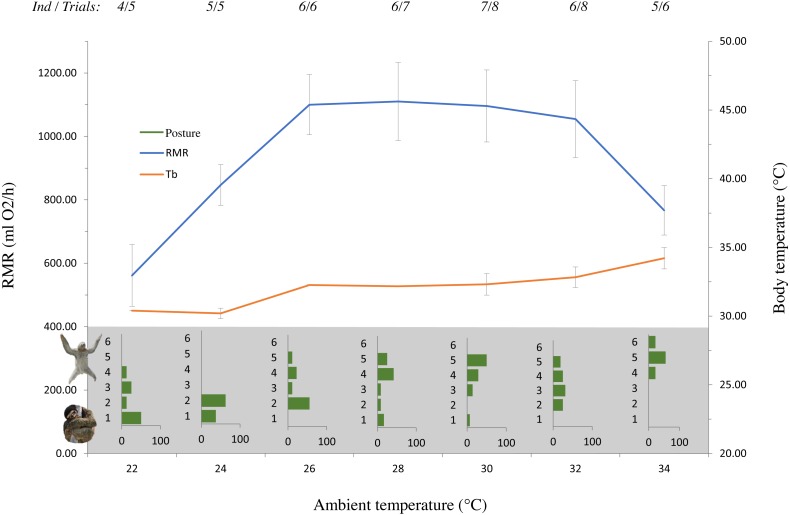
The effect of ambient temperature (*T*_*a*_) on resting metabolic rate (RMR), rectal temperature (*T*_*b*_) and posture of *Bradypus variegatus* sloths. Means presented (±1SE) are taken from 8 animals over a total of 10 different trials (repeated measurements for individual sloths). Number of individuals and trials at each temperature bracket listed across the top. Posture was graded visually on a scale of 1–6 (1 = tight ball, 6 = all limbs spread) and is presented as a frequency distribution with bars representing the proportion of cases. *T*_*a*_ significantly affected RMR, with the lowest metabolic values occurring at 22 °C (561 ml O_2_/h ± 95 ml O_2_/h). RMR increased with increasing *T*_*a*_, before peaking and remaining constant between 26–30 °C (1,102 ml O_2_/h ± 119 ml O_2_/h). Above 30 °C, RMR decreased rapidly. Both sloth *T*_*b*_ and posture were significantly affected by changes in *T*_*a*_. Photo credit: Rebecca Cliffe

Observations were made throughout each experimental run by looking through the peep hole in the water bath and recording a value every 4 min. Posture and activity were recorded on a scale of 1–6 (1 = tight ball/sleep, 6 = all limbs spread/vigorous activity) (Figs. 1 & S5)

### Statistical analyses

Statistical analyses were conducted in R (R Core Team, 2014). Shapiro–Wilk normality tests were completed on data to determine which statistical tests would be appropriate. The relationship between RMR and *T*_*a*_ was determined using a hierarchical linear mixed model (LMM) fitted using the ‘lmer’ from the “lme4” package with delta AICc model selection. Body mass, sex and captivity status were entered as covariates and animal ID as a random factor to allow for repeated measurements within individuals. Models were compared to determine *χ*^2^ and *p* values using likelihood ratio tests from the function ‘Anova’ in package “car”. When determining the effect of *T*_*a*_ on RMR, only data from the 10 trials in which sloths were exposed to a wide range of ambient temperatures were included in the analysis. We calculated the percentage error of the allometric predictions by dividing the difference between measured and allometric values by the allometric value. RMR values were compared with the allometric predictions using one-sample t-tests. The effect of *T*_*a*_ on *T*_*b*_ was examined using a generalized linear model (GLM), where *T*_*a*_ was in interaction with animal ID, and the significance of this term determined by comparing GLMs with and without this interactive term using an anova model comparison. The effect of *T*_*a*_ on posture and activity was examined using ordinal response regression models, with posture and activity as the dependant variables in each case. P-values were obtained using the function ‘Anova’ in package “car” which performs a Wald Chi-square test.

## Results

### Effect of *T*_*a*_ on Resting Metabolic Rate (RMR)

Means presented (±SE). Ambient temperature affected mean RMR (*χ*^2^(1) = 8.3095, *p* = 0.004), with the lowest metabolic rate (561 ml O_2_/h ± 95 ml O_2_/h) occurring at the lowest *T*_*a*_ (22 °C) (Fig. 1). As *T*_*a*_ increased, mean RMR values increased and remained high and constant between 26–30 °C (1,102 ml O2/h ± 119 ml O2/h), before decreasing thereafter (Fig. 1).

### Effect of *T*_*a*_ on *T*_*b*_ and posture

Body temperature was recorded over a maximum range (across all animals) of 4.7 °C. The average range of *T*_*b*_ for each individual sloth was 2.0 °C, from a minimum of 30.2 °C to a maximum of 34.9 °C and was significantly related to changes in ambient temperature (*χ*2(16, 3) = 0.885, *p* = 0.001). As with metabolic rate, *T*_*b*_ did not differ significantly and was constant between *T*_*a*_ values of 26 and 30 °C (Fig. 1). The overall thermal conductance was 0.3 ml O2/g h ° ± 0.4 ml O2/g.h ° (SD) (Fig. S6). Body posture was significantly related to *T*_*a*_ (*χ*2(1) = 11.313, *p* = 0.001), with the incidence of animals adopting spread-out postures increasing at higher temperatures.

### Effect of body mass and sex on RMR

Across the eight sloths, mean body mass was 3.9 kg ± 0.5 kg (SD) and overall mean RMR was 432 kJ/day ± 155 kJ/day (SD) (increasing to 488 kJ/day ± 180 kJ/day (SD) within the mid temperature range where RMR remains least variable [26–30 °C]) (Table S1). Mean RMR values within this stable mid-temperature range (kJ/day) were significantly lower than both of the allometric predictions by Kleiber (1961) (*M* = 816, *SD* = 85  *t*(13) =  − 7.546, *p* = 0.001) and White & Seymour (2003) (*M* = 581, *SD* = 55  *t*(13) =  − 2.345, *p* = 0.034).

Captivity status (wild vs captive sloths) and sex did not have a significant effect on sloth RMR (*χ*^2^(1) = 1.747, *p* = 0.186) (*χ*^2^(1) = 0.225, *p* = 0.636). However, our small sample size limits the power of this result. There was a significant effect of body mass on RMR within the mid-temperature range where RMR remains least variable (26–30 °C) (*y* =  − 69.632*x* + 398.96, *R*^2^ = 0.401, *p* = 0.020) where ×represents body mass (kg) and y represents RMR (kJ/kg.day).

## Discussion

Our results are broadly comparable to both the RMR and field metabolic rate (FMR) values previously recorded for three-fingered sloths (Irving, Scholander & Grinnell, 1942; McNab, 1978; Nagy & Montgomery, 1980; Pauli et al., 2016) and confirm the notion that sloths have one of the lowest metabolic rates of any non-hibernating mammal. Indeed, values of VO_2_ measured in the stable mid-temperature range (26–30 °C) were 40% lower, on average, than the prediction made by Kleiber (1961) for mammals, and 16% lower than the prediction of White & Seymour (2003), which takes into account the additional variation attributable to *T*_*b*_, phylogeny and digestive state (Table S1). There are multiple explanations as to why sloths have such a low metabolic rate, with perhaps the most popular being that their low-calorie diet which is high in toxicity, combined with an atypically long digestion period, means that they acquire energy too slowly to be able to expend it rapidly (Foley, Engelhardt & Charles-Dominique, 1995; Brian K. McNab, 1978; Montgomery & Sunquist, 1978; Nagy & Montgomery, 1980).

Although sloths have a RMR that falls significantly below predictions based on body mass, they are not unique among mammals in this aspect. In particular, several species of fossorial rodents (e.g., *Geomys pinetis, Spalax leucodon, Tachyoryctes splendens, Heliophobius kapeti, Heterocephalus glaber*) as well as the Himalayan red panda (*Ailurus fulgens*), binturong (*Arctictis binturong*) and giant panda (*Ailuropoda melanoleuca*) (McNab, 2005; McNab, 1988; Nie et al., 2015) are known to have a lower than expected rate of metabolism (Goldman et al., 1999; McNab, 1966). As for sloths, current explanations for this also relate to the low rates at which energy is acquired by these animals (Fei et al., 2016; McNab, 1978).

The notable difference in the response of sloths is that their metabolic response to changes in ambient temperature appears to be the inverse of that expected for a typical homeotherm. This response, coupled with an obvious plasticity in body temperature, (Britton & Atkinson, 1938; Irving, Scholander & Grinnell, 1942; Montgomery & Sunquist, 1978), contrasts to the highly stenothermal state for most non-hibernating mammals (Phuoc & Ngoan, 2005). In particular, the ‘inverted’ U-shape of the curve relating VO_2_ to *T*_*a*_ is highly unusual. It appears that sloths incorporate the drop in metabolic rate with *T*_*a*_ on the left hand side of the metabolic plateau (characteristic of poikilotherms), with a drop in metabolic rate with increasing temperature on the right-hand side of the plateau (which is characteristic of some homeotherms that engage in torpor, hibernation and aestivation Heldmaier, Ortmann & Elvert, 2004; McNab, 2002).

It would seem that sloths have limited capacity to produce heat at low *T*_*a*_ values (Britton & Atkinson, 1938; Montgomery & Sunquist, 1978) which, we suggest, leads to their reduced metabolic rates. While an unusual response for adult homeotherms, similar responses do occur in neonatal mammals which are not yet thermally independent (Mortola & Naso, 1998). Indeed, sloths have recently been found to have non-functional uncoupling protein 1 (UCP1) which is essential for non-shivering thermogenesis (Gaudry et al., 2016). It is notable, however, that the sloths did attempt to minimize heat loss by retracting their limbs and reducing the exposed surface area of their bodies. The nominal sloth ‘TAZ’, corresponding to their metabolic peak at 26–30 °C, coincides closely with average daytime temperatures in tropical forests (Giné et al., 2015), when sloths are most active and feed the most (Chiarello, 1998; Cliffe et al., 2015; Giné et al., 2015). At these temperatures, we expect heat production to exactly balance that lost due to the small difference in sloth body and environmental temperature (of some 4 °C). Indeed, the highly restricted distribution of sloths (Voss et al., 2009) places them in environments with stable ambient temperatures that deviate little from this range.

When *T*_*a*_ values exceeded 30 °C, sloths reduced their VO_2_ in a manner reminiscent of the fat-tailed dwarf lemur (*Cheirogaleus medius*), which reduces VO_2_ for months, undergoing hibernation in response to high and variable *T*_*a*_ values (Dausmann et al., 2004). In homeotherms, states of hibernation, torpor or aestivation are typically characterised by active depression of metabolic rate combined with associated decreases in *T*_*b*_ (Geiser, 2004; Geiser & Ruf, 1995). However, the underlying molecular mechanisms involved in these responses are still poorly understood and are likely to be multi-faceted (Rider, 2016). The ‘hibernator as neonate’ hypothesis suggests that the ability for homeothermic mammals to resume a heterothermic state as adults results from the continued expression of particular genes that are retained from the neonate form (Harris, Olson & Milsom, 1998). While sloths do show metabolic depression at high *T*_*a*_’s, there is no corresponding drop in *T*_*b*_ as would be expected for a mammal entering torpor or aestivation, and the animals were not apparently in a distinct state of inactivity during this period compared to other *T*_*a*_’s (Fig. S5). In a number of homeotherms, sleep and activity state have been shown to account for variation in the metabolic response to temperature due to the inhibition of thermogenic responses (Glotzbach & Craig Heller, 1984; Heller et al., 2011; Heller & Glotzbach, 1977). Although there were no notable differences in sloth activity across the different temperature brackets (Fig. S5), state may account for some of the observed intra-individual variation in metabolic rate (Table S1). To our knowledge, this is the first observation of a mammal temporarily, and ostensibly strategically, depressing metabolic activity as a direct response to high ambient temperatures, without entering into states of torpor, aestivation or hibernation. The concomitant adoption of a more ‘spread-eagled’ body posture may serve to facilitate heat loss in a manner seen in more conventional mammals (Briscoe et al., 2014). Ultimately this broadens our knowledge of how animals deal with variation in temperatures, and further work to determine the underlying molecular mechanisms controlling the metabolic depression in sloths could provide important insights into the active control and suppression of metabolic rate in all mammals.

## Conclusions

We suggest that sloths depress VO_2_ at higher *T*_*a*_ values in order to prevent hyperthermia. Due to slow rates of digestion limiting the rates of energy acquisition (Cliffe et al., 2015; Foley, Engelhardt & Charles-Dominique, 1995), all sloths are considered to exist under severe energetic constraints (Pauli et al., 2016). Delicate adjustments of metabolic rate—in part as a response to *T*_*a*_—are one way in which sloths adjust and minimise their energy expenditures. The apparent relaxed homeothermy of sloths would therefore seem to incorporate metabolic depression as an effective strategy to prevent uncontrolled escalations in both *T*_*b*_ and consequently energy expenditure under hot environmental conditions. Reductions in VO_2_ therefore serve both to minimise energy expenditure at *T*_*a*_’s below the ‘TAZ’ and to reduce the risk of hyperthermia above the ‘TAZ’.

##  Supplemental Information

10.7717/peerj.5600/supp-1 Supplemental Information 1 Supplementary materials

10.7717/peerj.5600/supp-2 Figure S1 The change in body temperature over time during cold exposure for 2 *B. variegatus* sloths Rectal temperature was recorded at 30-minute intervals with the metabolic chamber maintained between 17–19 °C. 92% (sloth A) and 86% (sloth B) of total body temperature cooling occurred within the first 60 minutes after the animal entered the chamber.

10.7717/peerj.5600/supp-3 Figure S2 Difference between ambient temperature and core body temperature (temperature differential, °C) against the rate of change of core body temperature (°C/min) for two *B. variegatus* sloths, *A* and *B* (cf. Fig. S1)

10.7717/peerj.5600/supp-4 Figure S3 The stabilisation of oxygen and carbon dioxide levels over time Gas concentration (%) values were recorded every 2 minutes from one adult *B.variegatus* sloth inside the metabolic chamber at 29 °C. The mean sloth respiratory quotient (RQ) for the equilibrated data was 0.99 (± 0.01 SD).

10.7717/peerj.5600/supp-5 Figure S4 Diagram showing the mean temperature values (°C) and standard deviation (SD) at 12 different locations within the metabolic chamber during control tests Means taken from an empty chamber over 3 separate tests at 5 different temperature brackets.

10.7717/peerj.5600/supp-6 Figure S5 Sloth activity levels at different ambient temperature brackets ****Data are taken from 8 animals over a total of 10 different trials (repeated measurements for individual sloths). Activity was graded visually on a scale of 1–6 (1 = sleep, 6 = vigorous activity) and is presented as a frequency distribution with bars representing the proportion of cases. There was no significant effect of *T*_*a*_ on sloth activity levels (*χ*2(1) = 0.093, *p* = 0.7609).

10.7717/peerj.5600/supp-7 Figure S6 Thermal conductance and posture of sloths (*Bradypus variegatus*) at different ambient temperatures Data are taken from 4 animals over 4 trials. Posture was graded visually on a scale of 1–6 (1 = tight ball, 6 = all limbs spread).

10.7717/peerj.5600/supp-8 Figure S7 Resting metabolic rate (RMR) of 2 *B. variegatus* sloths at various times of the day at constant ambient temperatures (*T*_*a*_)

10.7717/peerj.5600/supp-9 Figure S8 The effect of ambient temperature on the metabolic rate of 4 *B. variegatus* sloths (A, B, C and D) during individual runs in the metabolic chamber Additional body temperature data shown for sloths *c* and *d*. Data taken from trials 1/a, 5/a, 4/b and 6/c (see Table S1).

10.7717/peerj.5600/supp-10 Table S1 Body mass, RMR, allometric predictions and body temperature data for 8 *B. variegatus* sloths

10.7717/peerj.5600/supp-11 Data S1 Raw data of metabolic rate versus temperature

## Additional Information and Declarations

### Data Availability

The following information was supplied regarding data availability: The raw data are provided in a Supplemental File.
