# Supplementary material for: The metabolic response of the Bradypus sloth to temperature
Source: PeerJ. 2018 Sep 19;6:e5600. doi: 10.7717/peerj.5600 (PMC6151113; doi:10.7717/peerj.5600)
Supplement: Supplemental Information 1 [file peerj-06-5600-s001.docx]

**The metabolic response of the *Bradypus* sloth to temperature**

SUPPLEMENTARY INFORMATION

***Preliminary work***

An initial study was conducted to determine the length of time required for sloth rectal temperature to equilibrate sufficiently with the chamber temperature. For this, two habituated sloths were taken from their normal ambient temperatures (ca. 28°C) and placed in the metabolic chamber at the coldest temperature (ca. 18°C) while their rectal temperature was measured. In these cases, we noted that it took between 90 and 120 minutes for 99% of the total body temperature change to occur in the two sloths, although 90% of cooling was observed to occur, for both individuals, within the first hour (Figs. S1 and S2).

To ensure there were no time-based differences in RMR (e.g. for chronobiological reasons), two sloths were maintained in the chamber at a constant temperature until oxygen and carbon dioxide levels had stabilised. This was repeated for both animals at temperatures within the thermoneutral zone at various times of the day (between 8am and 9pm) (Fig. S6).

***Habituation and stress***

Due to repeated experimental runs with some animals, there is a possibility that some level of habituation might occur with the respirometry chamber. Nevertheless, we suggest that any effects of this are minimal as the sloths that were tested on more than one occasion did not show any reduced metabolic rate measurements or respiration rate on either the second or third times exposed to the chamber (see Table S1).

**Fig. S1.**

**The change in body temperature over time during cold exposure for 2 *B. variegatus* sloths.** Rectal temperature was recorded at 30-minute intervals with the metabolic chamber maintained between 17-19°C. 92% (sloth *A*) and 86% (sloth *B*) of total body temperature cooling occurred within the first 60 minutes after the animal entered the chamber.

**Fig. S2**

**Difference between ambient temperature and core body temperature (temperature differential, °C) against the rate of change of core body temperature (°C/min) of two *B. variegatus* sloths, *A* and *B.***

**Fig. S3**

**The stabilisation of oxygen and carbon dioxide levels over time.** Gas concentration (%) values were recorded every 2 minutes from one adult *B. variegatus* sloth inside the metabolic chamber at 29°C. The mean sloth respiratory quotient (RQ) for the equilibrated data was 0.99 (±0.01 SD).

**Fig. S4.**

**Diagram showing the mean temperature values (°C) and standard deviation (SD) at 12 different locations within the metabolic chamber during control tests.** Means taken from an empty chamber over 3 separate tests at 5 different temperature brackets.

**Fig. S5.**

**Sloth activity levels at different ambient temperature brackets.** Data are taken from 8 animals over a total of 10 different trials (repeated measurements for individual sloths). Activity was graded visually on a scale of 1-6 (1 = sleep, 6 = vigorous activity) and is presented as a frequency distribution with bars representing the proportion of cases. There was no significant effect of T_a_ on sloth activity levels (χ2 (1) = 0.093, p = 0.7609).

**Fig. S6.**

**Thermal conductance and posture of sloths (*Bradypus variegatus*) at different ambient temperatures.** Data are taken from 4 animals over 4 trials. Posture was graded visually on a scale of 1-6 (1 = tight ball, 6 = all limbs spread).

**Fig. S7.**

**Resting metabolic rate (RMR) of 2 *B. variegatus* sloths at various times of the day at constant ambient temperatures (T_a_).**

**Fig. S8.**

**The effect of ambient temperature on the metabolic rate of 4 *B. variegatus* sloths (*a*, *b, c* and *d)* during individual runs in the metabolic chamber.** Additional body temperature data shown for sloths *c* and *d*. Data taken from trials 1/a, 5/a, 4/b and 6/c (see table S1).

**Table S1.**

**Body mass, RMR, allometric predictions and body temperature data of 8 *B. variegatus* sloths.**
