# Supplementary figures and images for: The metabolic response of the Bradypus sloth to temperature
Source: PeerJ. 2018 Sep 19;6:e5600. doi: 10.7717/peerj.5600 (PMC6151113; doi:10.7717/peerj.5600)

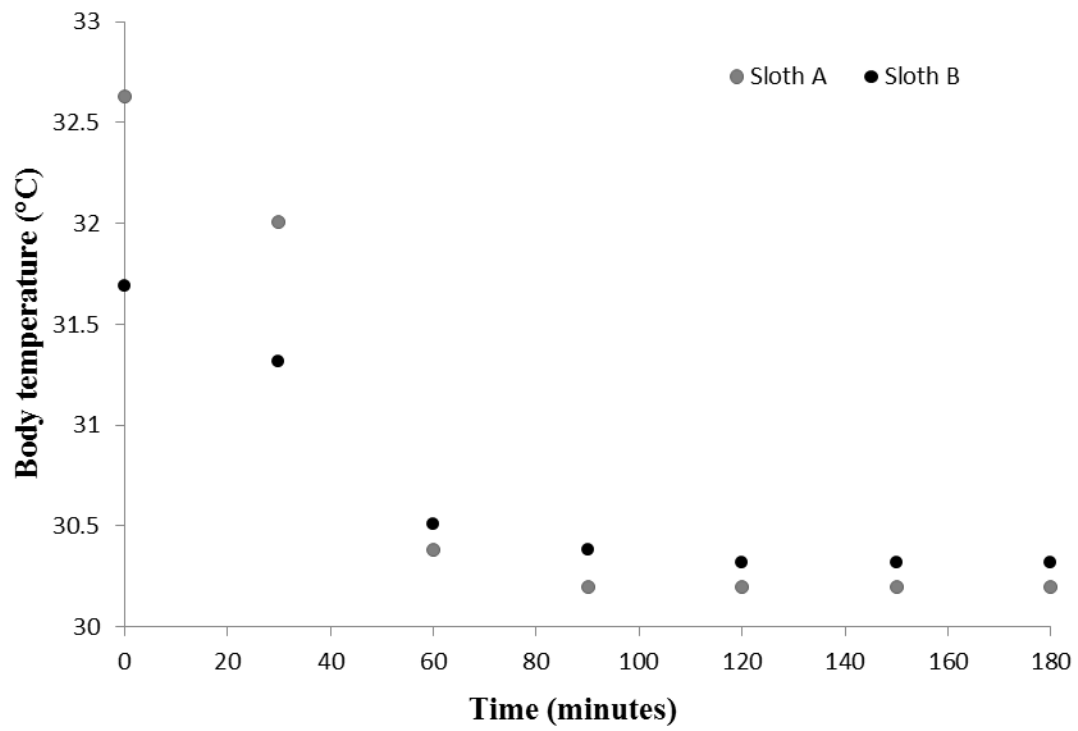

Supplement: Figure S1 — Rectal temperature was recorded at 30-minute intervals with the metabolic chamber maintained between 17–19 °C. 92% (sloth A) and 86% (sloth B) of total body temperature cooling occurred within the first 60 minutes after the animal entered the chamber. [file peerj-06-5600-s002.pdf]

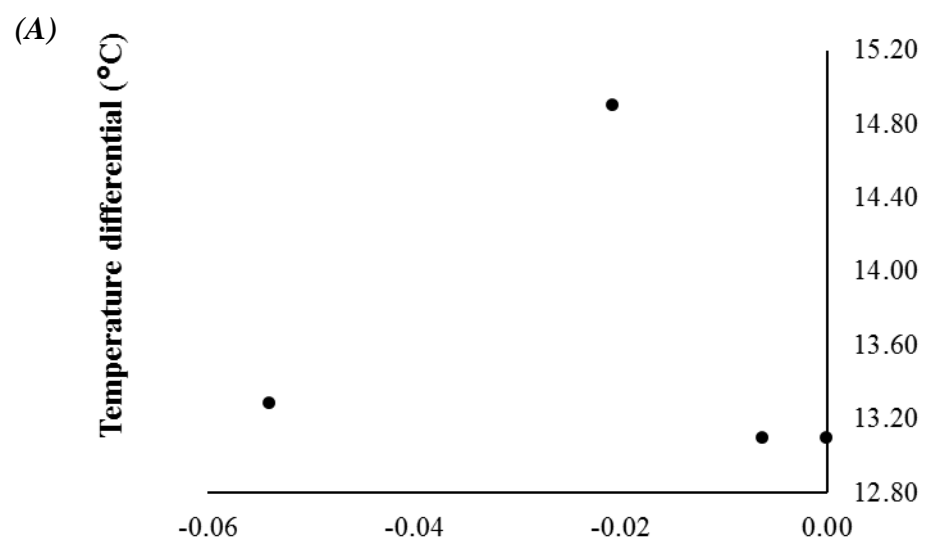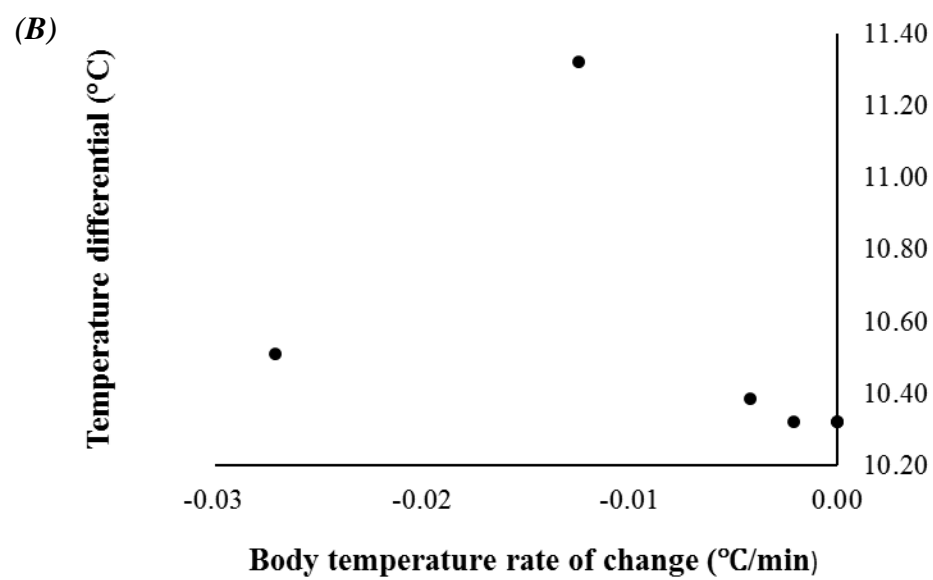

Supplement: Figure S2 [file peerj-06-5600-s003.pdf]

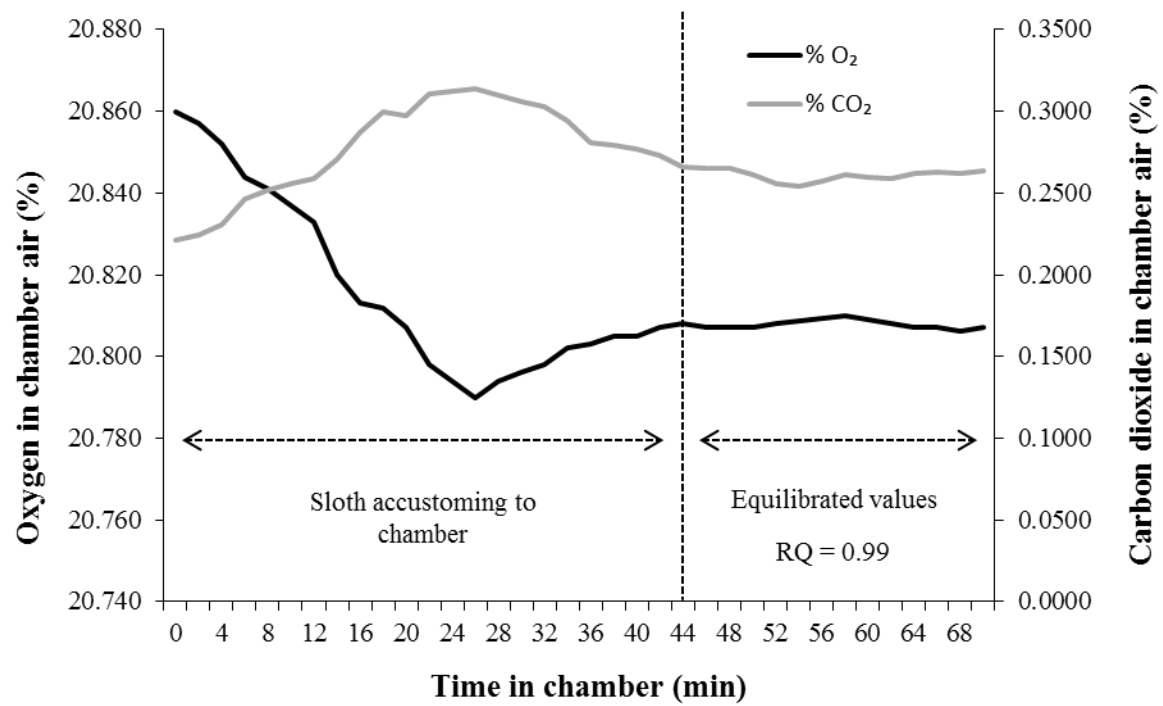

Supplement: Figure S3 — Gas concentration (%) values were recorded every 2 minutes from one adult B.variegatus sloth inside the metabolic chamber at 29 °C. The mean sloth respiratory quotient (RQ) for the equilibrated data was 0.99 (± 0.01 SD). [file peerj-06-5600-s004.pdf]

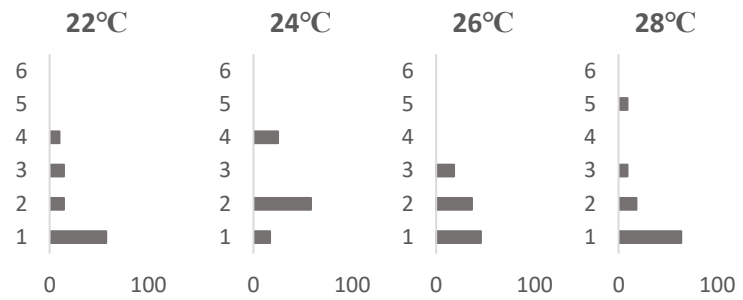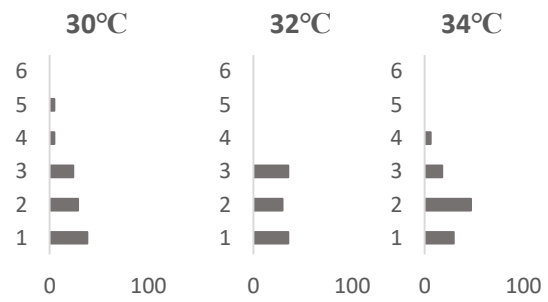

Supplement: Figure S5 — Data are taken from 8 animals over a total of 10 different trials (repeated measurements for individual sloths). Activity was graded visually on a scale of 1–6 (1 = sleep, 6 = vigorous activity) and is presented as a frequency distribution with bars representing the proportion of cases. There was no significant effect of Ta on sloth activity levels (χ2(1) = 0.093, p = 0.7609). [file peerj-06-5600-s006.pdf]

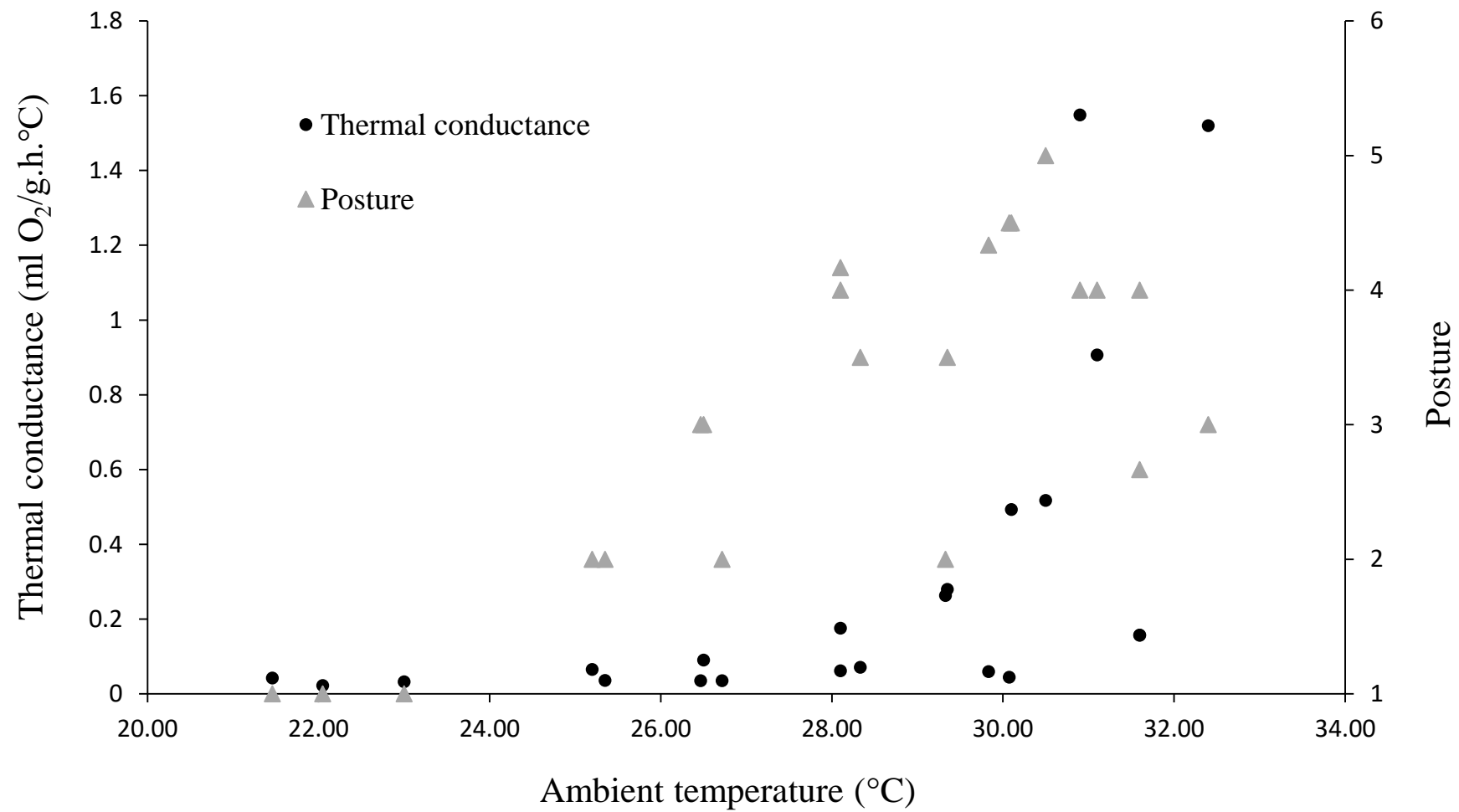

Supplement: Figure S6 — Data are taken from 4 animals over 4 trials. Posture was graded visually on a scale of 1–6 (1 = tight ball, 6 = all limbs spread). [file peerj-06-5600-s007.pdf]

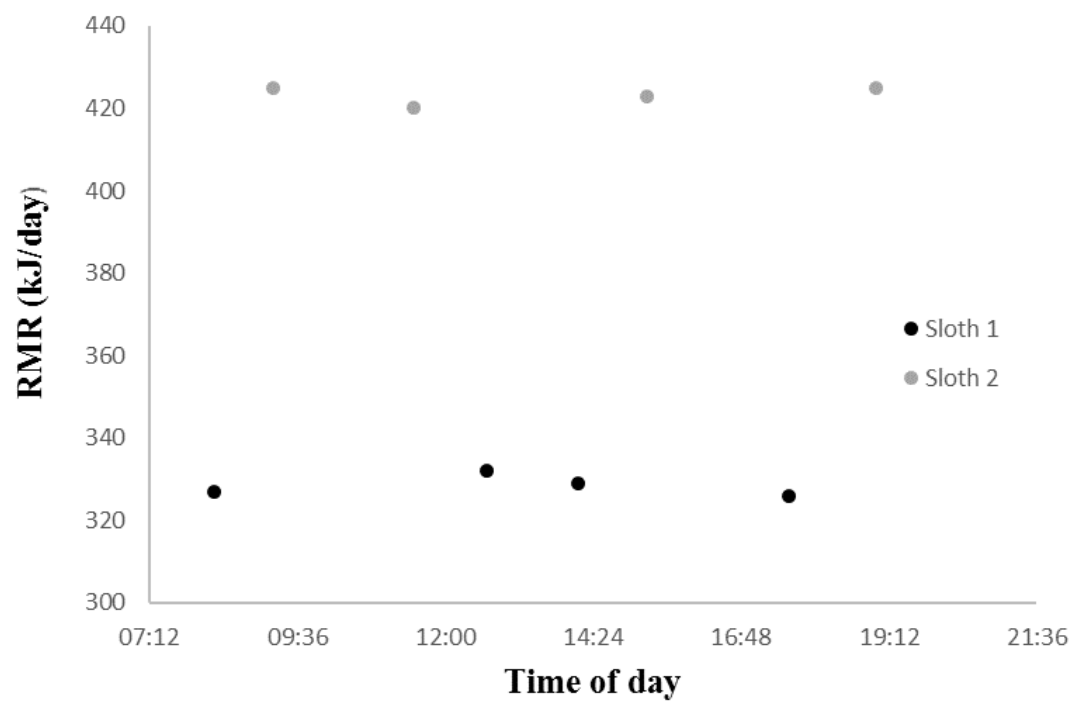

Supplement: Figure S7 [file peerj-06-5600-s008.pdf]

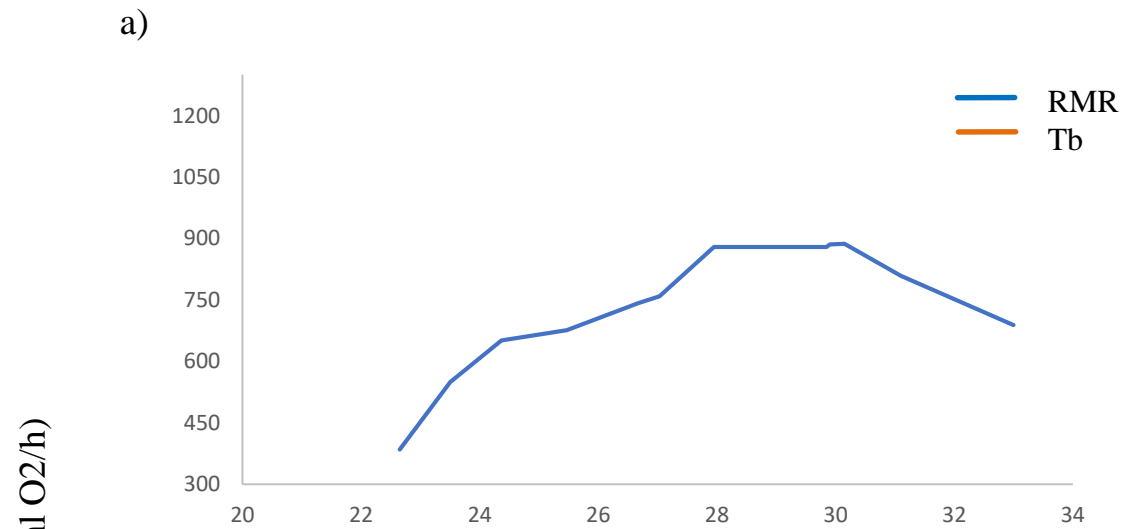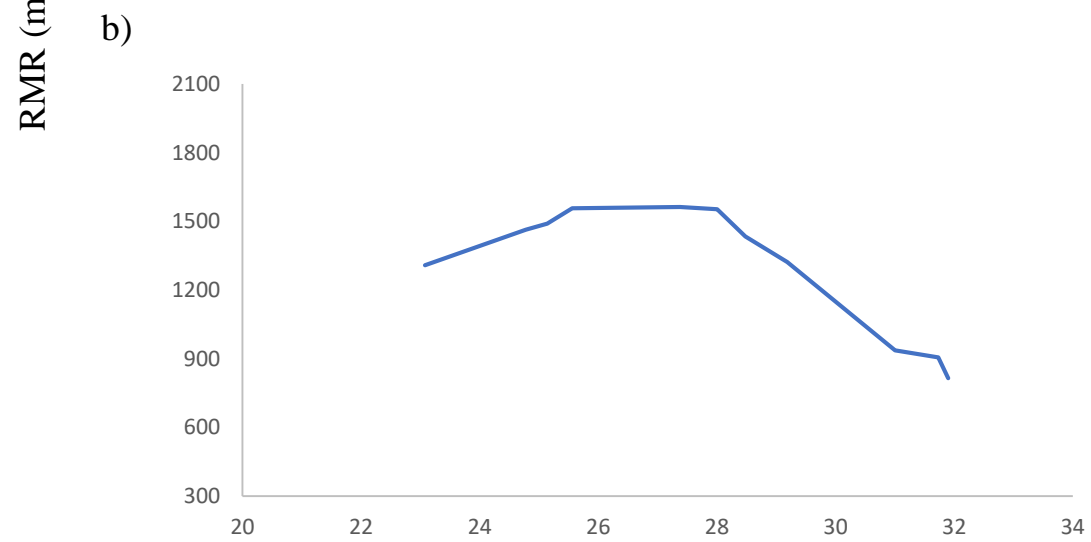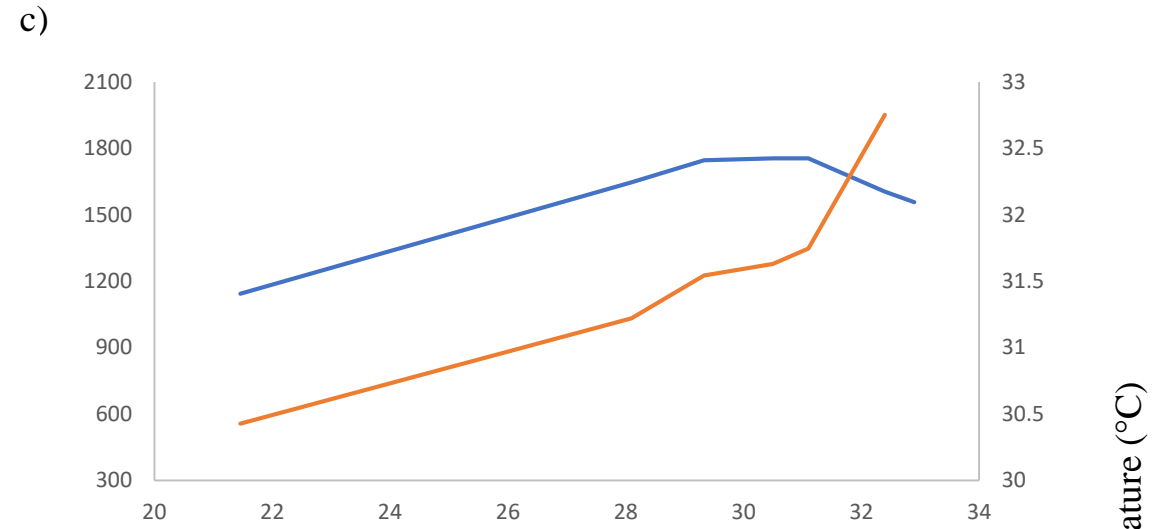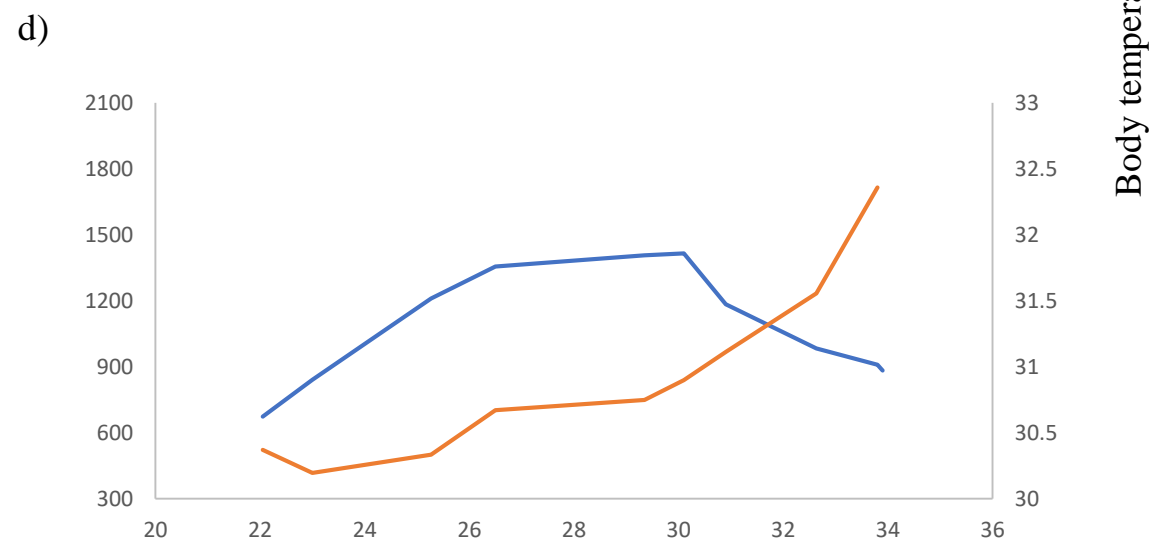

Ambient temperature (°C)

Supplement: Figure S8 — Additional body temperature data shown for sloths c and d. Data taken from trials 1/a, 5/a, 4/b and 6/c (see Table S1). [file peerj-06-5600-s009.pdf]
