# Supplementary material for: The metabolic response of the Bradypus sloth to temperature
Source: PeerJ. 2018 Sep 19;6:e5600. doi: 10.7717/peerj.5600 (PMC6151113; doi:10.7717/peerj.5600)
Supplement: Figure S4 — Means taken from an empty chamber over 3 separate tests at 5 different temperature brackets. [file peerj-06-5600-s005.pdf]

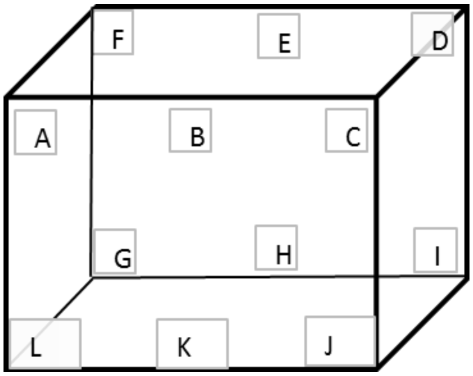

Chamber location	Temperature bracket (°C)									
	**18-19**	**SD**	**21-22**	**SD**	**26-27**	**SD**	**29-30**	**SD**	**32-34**	**SD**
**A**	19.30	0.35	22.20	0.17	26.30	0.10	29.23	0.74	32.23	0.21
**B**	19.23	0.32	22.20	0.10	26.23	0.12	29.23	0.80	32.23	0.12
**C**	19.27	0.31	22.07	0.15	26.23	0.06	29.37	0.75	32.17	0.35
**D**	19.30	0.40	22.10	0.00	26.13	0.29	29.10	0.60	31.97	0.32
**E**	19.27	0.38	22.10	0.10	26.07	0.32	29.37	0.76	32.17	0.32
**F**	19.17	0.25	22.03	0.15	26.10	0.30	29.17	0.67	32.17	0.32
**G**	17.07	0.35	21.33	0.12	26.30	0.10	27.43	0.76	32.33	0.31
**H**	17.13	0.67	21.37	0.15	26.30	0.20	27.80	0.36	32.43	0.40
**I**	17.10	0.52	21.23	0.21	26.27	0.35	27.33	1.06	32.37	0.25
**J**	17.13	0.58	21.37	0.06	26.27	0.12	27.47	0.35	32.70	0.17
**K**	17.13	0.67	21.20	0.26	26.23	0.12	27.97	0.40	32.43	0.06
**L**	16.73	0.23	21.20	0.17	26.17	0.06	27.57	0.50	32.20	0.26
