# Supplementary material for: The metabolic response of the Bradypus sloth to temperature
Source: PeerJ. 2018 Sep 19;6:e5600. doi: 10.7717/peerj.5600 (PMC6151113; doi:10.7717/peerj.5600)
Supplement: Table S1 [file peerj-06-5600-s010.docx]

| **Sloth/trial** | **Trial start time** | **Total length of trial** |  | **Sex** | **Mass (kg)** | **RMR** | | | | | **T_a_ range (°C)** | **T_b_ range (°C)** | **RMR range (kJ/day)** |  |
| --- | --- | --- | --- | --- | --- | --- | --- | --- | --- | --- | --- | --- | --- | --- |
|  |  |  | **Number of measurements** |  |  | **(Kcal/**  **day)** | **(kJ/**  **day)** | **RQ** | **Kleiber prediction**  **(Kcal/day)** | **White and Seymour prediction (Kcal/day)** |  |  |  | **Average respiration rate (bpm)**  **± SD** |
| 1/a | 12:34 | 7h 45m | 7 | M | 3 | 192.12 | 772.34 | 0.75 | 159.57 | 115.81 | 21-33 | 30.4-32.8 | 551-846 | 8 ± 2 |
| 2/a | 16:20 | 3h 20m | 3 | M | 3.9 | 94.22 | 378.78 | 1.01 | 194.27 | 138.43 | 27 | - | 358-404 | 8 ± 3 |
| 2/b | 14:20 | 8h 40m | 8 | M | 3.9 | 86.91 | 349.38 | 0.85 | 194.27 | 138.43 | 23-33 | - | 327-365 | 10 ± 2 |
| 2/c | 10:19 | 6h | 5 | M | 4 | 164.07 | 659.56 | 0.78 | 197.99 | 140.84 | 25-32 | 33.4-34.0 | 565-709 | 10 ± 2 |
| 3/a | 09:30 | 2h | 2 | F | 4.4 | 88.09 | 354.14 | 1.00 | 212.66 | 150.27 | 28 | - | 267-441 | 11 ± 1 |
| 3/b | 13:00 | 7h | 5 | F | 5.1 | 77.24 | 310.51 | 0.92 | 237.56 | 166.14 | 23-33 | - | 247-467 | 11 ± 2 |
| 4/a | 14:55 | 3h 40m | 2 | F | 3.9 | 106.69 | 428.90 | 0.97 | 194.27 | 138.43 | 28 | - | 422-435 | 13 ± 4 |
| 4/b | 10:26 | 11h 30m | 11 | F | 3.8 | 156.50 | 629.11 | 0.90 | 190.52 | 136.01 | 23-32 | - | 393-753 | 12 ± 5 |
| 5/a | 08:50 | 12h | 12 | M | 4 | 87.94 | 353.50 | 0.91 | 197.99 | 140.84 | 22-33 | - | 185-428 | 9 ± 3 |
| 6/a | 09:33 | 3h 20m | 3 | F | 3.3 | 69.50 | 279.41 | 0.91 | 171.39 | 123.57 | 23 | - | 138-393 | 10 ± 5 |
| 6/b | 11:04 | 7h 40m | 7 | F | 4.4 | 83.08 | 333.99 | 0.98 | 212.66 | 150.27 | 22-33 | - | 156-449 | 8 ± 3 |
| 6/c | 11:20 | 10h 15m | 10 | F | 3.6 | 130.34 | 523.96 | 0.80 | 182.95 | 131.10 | 22-34 | 30.2-32.4 | 324-683 | 9 ± 2 |
| 7/a | 14:48 | 4h | 4 | F | 3.1 | 64.00 | 257.30 | 0.89 | 163.54 | 118.42 | 25-29 | - | 102-398 | 12 ± 4 |
| 7/b | 09:00 | 10h 30m | 10 | F | 3.8 | 79.99 | 321.55 | 0.90 | 190.52 | 136.01 | 21-34 | - | 214-410 | 8 ± 2 |
| 8/a | 11:29 | 8h | 4 | M | 4.4 | 129.82 | 521.87 | 0.82 | 212.66 | 150.27 | 26-30 | 33.4-34.9 | 457-595 | 16 ± 3 |
| **Mean** |  |  |  |  | **3.91** | **107.37** | **431.62** | **0.89** |  |  |  |  |  |  |
